# Impaired emotion recognition is associated with sleep-related hypoxic burden

**DOI:** 10.1093/sleep/zsag078

**Published:** 2026-05-28

**Authors:** Dimitri Ferretti, Elena Richert, Ding Zou, Jan Hedner, Christian Strassberger, Erna Sif Arnardottir, Ludger Grote, Kamilla Rún Jóhannsdóttir

**Affiliations:** Department of Psychology, Reykjavik University, Reykjavik, Iceland; Reykjavik University Sleep Institute, Reykjavik University, Reykjavik, Iceland; Department of Internal Medicine and Clinical Nutrition, Sahlgrenska Academy, University of Gothenburg, Gothenburg, Sweden; Department of Psychology, Reykjavik University, Reykjavik, Iceland; Reykjavik University Sleep Institute, Reykjavik University, Reykjavik, Iceland; Department of Technical Physics, University of Eastern Finland, Kuopio, Finland; Department of Internal Medicine and Clinical Nutrition, Sahlgrenska Academy, University of Gothenburg, Gothenburg, Sweden; Department of Internal Medicine and Clinical Nutrition, Sahlgrenska Academy, University of Gothenburg, Gothenburg, Sweden; Pulmonary Department, Sahlgrenska University Hospital, Gothenburg, Sweden; Department of Internal Medicine and Clinical Nutrition, Sahlgrenska Academy, University of Gothenburg, Gothenburg, Sweden; Reykjavik University Sleep Institute, Reykjavik University, Reykjavik, Iceland; Department of Computer Science, Reykjavik University, Reykjavik, Iceland; Department of Engineering, Reykjavik University, Reykjavik, Iceland; Department of Internal Medicine and Clinical Nutrition, Sahlgrenska Academy, University of Gothenburg, Gothenburg, Sweden; Pulmonary Department, Sahlgrenska University Hospital, Gothenburg, Sweden; Department of Psychology, Reykjavik University, Reykjavik, Iceland; Reykjavik University Sleep Institute, Reykjavik University, Reykjavik, Iceland

**Keywords:** cognition, emotion recognition, hypoxic burden, obstructive sleep apnea, Penn emotion recognition task, sleep-disordered breathing, sleep fragmentation

## Abstract

**Study Objectives:**

To identify predictors of emotion recognition performance from sleep metrics, including obstructive sleep apnea (OSA) and sleep architecture.

**Methods:**

Adults from the Icelandic population completed the Penn Emotion Recognition Task, assessing facial expressions presented with different intensities. Subsequently, participants underwent three consecutive nights with self-applied polysomnography. Sleep parameters were averaged across nights. Regression analyses, adjusted for age, gender, and depressive symptoms were used to examine associations between sleep and emotion recognition.

**Results:**

Fifty-five participants (47.3% males, mean age 46.4 ± 14.4 years, body mass index 27.9 ± 4.6 kg/m^2^, apnea-hypopnea index (AHI)15.1 ± 15.6 events/h, AHI ≥ 5 in 65%) completed the study. Mean reaction times (ms) were 2746 ± 1294 for high intensity emotion, 2786 ± 995 for low intensity emotion, and 3308 ± 2192 for neutral stimuli, while recognition accuracy was 88.8 ± 8.9% for high intensity emotion, 73.3 ± 10.2% for low intensity emotion, and 85.0 ± 19.2% for neutral stimuli. Hypoxic burden metrics and sleep architecture, particularly desaturation severity (B = 363 ms for low intensity emotion, *p* < .01), sleep efficiency (B = −335 ms for low *p* = .01, B = −415 ms for high intensity emotion *p* = .02) and REM percentage (B = −310 ms for low intensity, *p* = .02), independently predicted performance. Using multiple-night polysomnography improved the strength of the linear models compared to data from a single-night.

**Conclusions:**

OSA-related hypoxic burden and sleep architecture are significantly associated with emotion recognition, underlying the importance of sleep for neurocognitive vulnerability.

**Statement of Significance**

The impact of obstructive sleep apnea (OSA) on cognitive performance has been studied. However, little is known about its effects on emotion recognition, a key prosocial skill essential for interactions and psychological well-being. This study addresses this gap by focusing on general population with mild-to-severe OSA. It highlights the importance of using novel metrics of oxygen desaturation burden rather than the apnea-hypopnea index, which may underestimate the cognitive consequences of OSA. Other important sleep quality measures include sleep length and continuity. Future research should investigate the effects of improved sleep and OSA treatment on emotion recognition. The importance of understanding the subtle impact of OSA on emotion recognition is evident, as it is a crucial determinant of everyday social interaction.

## Graphical Abstract

**Graphical Abstract:**
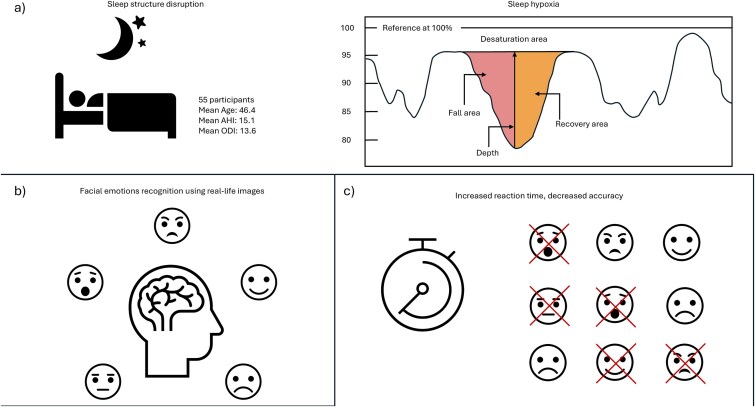

## Introduction

Obstructive sleep apnea (OSA) is recognized as the most prevalent sleep-related breathing disorder, affecting more than one billion adults worldwide [1]. OSA is characterized by recurrent episodes of complete or partial upper airway obstruction and increased respiratory effort during sleep, leading to decreased oxygen saturation (SpO_2_) and disrupted sleep architecture [2]. While OSA is well known for its negative effects on cardiovascular and metabolic health, growing evidence also points to a significant impact of OSA on impaired cognitive functioning.

Prior work on OSA has primarily focused on selective cognitive domains such as attention (vigilance), memory, and executive functions [3], whereas the effects of OSA on social cognition, particularly emotion processing, remain underexplored. This represents a critical gap in the literature, as emotion recognition plays a central role in social interaction and bonding, as well as general emotional well-being [4]. A limited number of studies report an impairment in recognizing facial or vocal emotional cues in individuals with OSA [5–7], suggesting that the disorder may interfere with the neural systems underlying affective processing. However, the magnitude and mechanisms linking OSA to emotion recognition and related socio-emotional deficits remain incompletely understood.

Evidence suggests that sleep fragmentation and conventional indices of OSA severity, such as the apnea-hypopnea index (AHI), impair emotion recognition [6]. However, to the author’s knowledge, no research has examined the role of hypoxic burden, although studies suggest that hypoxic burden may produce structural atrophy and hypoconnectivity in key emotion-processing regions in the brain, including the basolateral amygdala and insula areas [8, 9]. Furthermore, no study investigating facial emotion recognition in OSA has yet addressed multiple-night home sleep assessments. Prior work has used single-night, in-lab protocols, which can lead to inaccuracy in the sleep assessment by first-night effects and sleep disruption due to unfamiliar environments [10].

The main aim of the present study is to gain a better understanding of the potential OSA-emotion processing relationship by investigating the impact of: (1) hypoxic burden metrics, (2) AHI, and (3) sleep-architecture parameters on facial emotion recognition abilities (reaction time and accuracy). By evaluating these physiological and sleep-structure measures side by side, we seek to clarify the unique contribution of each parameter to social-cognitive functioning in individuals with mild-to-severe OSA. We hypothesized that hypoxic burden metrics and sleep architecture parameters (e.g. percentage of REM sleep stage (REM%), sleep efficiency, and wake after sleep onset (WASO)) would be better predictors of emotion recognition than standard respiratory-related parameters like the AHI.

## Materials and Methods

### Participants and demographics

Participants (*n* = 55, mean age 46.4 ± 15.0 years, 26 males (47.3%), mean body mass index (BMI) = 27.9 ± 4.6 kg/m^2^) were selected from a larger pool of individuals recruited by advertisement from the general Icelandic population. Participants completed an online screening questionnaire regarding comorbidities and sleep complaints. Being under treatment for OSA was an exclusion criterion for taking part in the study. The final analysis cohort comprised individuals with complete data sets, including self-applied polysomnography (PSG) studies (at least one valid night), neuropsychological assessment (The Depression, Anxiety and Stress Scale—21 Items, DASS-21) [11, 12], and a measure of emotion recognition by means of measurements (reaction time and accuracy) of the PENN Emotion Recognition Task (ER-40) [13]. Individuals were classified as OSA patients when AHI was at least 5 events/h (mean from multiple nights when applicable).

### Procedure

On the morning of the first sleep study, participants arrived at the research facilities at Reykjavik University. Trained personnel obtained written informed consent and collected anthropometric data, including height and weight measurements. Participants completed the ER-40. They were then provided with the self-applied PSG equipment and instructed to apply the PSG equipment to themselves for three consecutive nights before sleep. Upon completion of the recordings, the equipment was returned to the study center, where the data were downloaded and scored by an expert sleep technologist.

### Assessment of sleep

Sleep was assessed using self-applied home PSG for three consecutive nights. The PSG system (SAS, NOX A1S, NOX Medical, Reykjavik, Iceland) uses electrodes for frontal electroencephalography, electrooculography, electrocardiography, and leg electromyography [14]. Respiration during sleep is assessed by flow using a nasal cannula and respiratory movements by means of two respiratory inductance plethysmography belts. Finger pulse oximetry (Nonin Medical) assessed pulse rate and oxygen saturation.

The PSGs were scored in accordance with the American Academy of Sleep Medicine (AASM) manual, Version 2.6, by a single scorer supported by Noxturnal, software version 6.2.2, automated sleep and respiratory analysis [15]. AASM manual scoring rules were adapted according to the results of a previous self-applied PSG validation study [16]. Variables outside routine PSG capturing in detail the hypoxic load during sleep were derived from a publicly available software package (automatic blood oxygen saturation analysis software, ABOSA) and automatically calculated [17]. A detailed description of each parameter is presented in Table 1 [17–20].

**Table 1:** Description of variables calculated with ABOSA software

**Variable name**	**Description**
Desaturation Severity	The sum of desaturation areas normalized by the total sleep time [18]
Desaturation Duration	The percentage of the total duration of desaturations from total sleep time [19]
Recovery Severity	Same as desaturation severity but only for the recovery areas of the complete desaturation [20]
Recovery Duration	The duration of the recovery events following desaturation duration [17]
Recovery Index	Comparable to oxygen desaturation index but for the recovery areas only. It is always equal or smaller than ODI, as some desaturations stays at the lowest point meaning that there is no recovery back to baseline [20]

Table showing the name and description of variables calculated with ABOSA software and included in the data analysis.

### Emotion recognition measures, anthropometric and psychological assessment

Emotion recognition data were collected on the same morning as the anthropometric data. The ER-40 test was administered using Software Inquisit 6 (version 6.5.1, year 2021) in a room with no windows, a privacy screen between desks, and noise-cancelling headphones to minimize distractions. A total of up to three people were allowed to take the neurocognitive battery at the same time, and trained personnel were present to provide assistance if needed during the whole session.

**Figure 1:**
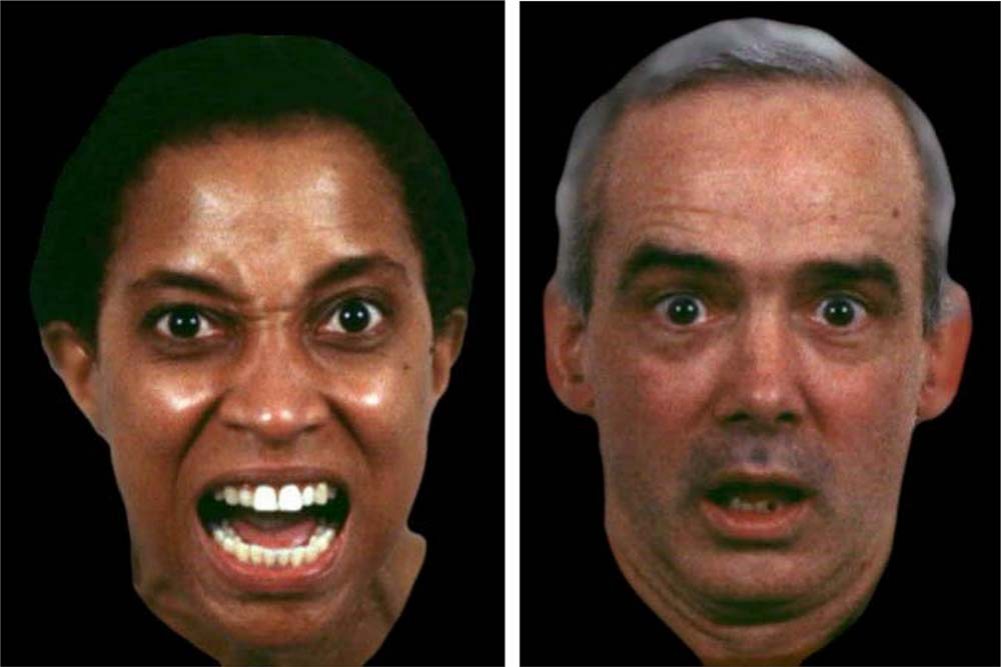
Exemplification of face recognition in the ER-40 test, example of the stimuli presented to participants. On the left panel, an example of high intensity anger is presented, whereas on the right panel, a stimulus of low intensity fear is shown.

The ER-40 comprises 40 photographs of human faces (20 males and 20 females, balanced for age and ethnicity in addition to gender, emotion and intensity) depicting five emotional expressions: happiness, sadness, anger, fear, and neutral as exemplified in Figure 1 [21, 22]. Each emotion category, except for neutral, is represented at both high and low intensities (*n* = 4 per intensity level, for a total of 16 high and 16 low intensity emotion stimuli). Stimuli were presented in random order. The task began with a practice trial during which participants received immediate feedback and were required to select the correct emotion before proceeding. During the test phase, participants were instructed to identify the emotion expressed in each face by choosing one of five response options (sad, happy, anger, fear, no emotion). Performance data were collected for both accuracy (percentage of correct or incorrect responses) and reaction time (measured in milliseconds, ms). Once the data collection ended, the answers were grouped as high-intensity, low-intensity, and neutral emotion stimuli.

DASS-21 is a validated psychological assessment instrument to detect self-reported symptoms of depression, stress, and anxiety [23]. The instrument consists of 21 items, 7 for each of the three subscales (depression, anxiety, stress). Each item is a statement connected to 4-point Likert scale (0 “did not apply to me”, 1 “applied to me to some degree or some of the time”, 2 “applied to me to a considerable degree or a good part of time”, 3 “applied to me very much or most of the time”). The score is calculated for each scale by summing the points related to the relevant items and then multiplying them by two. For each subscale, the results can range between five levels (normal, mild, moderate, severe, extremely severe) of severity, with scores ranging between 0 and 42.

### Data analysis

#### Descriptive statistics

Descriptive statistics were used to analyze demographic characteristics and PSG variables. Ordinal numerical variables were presented as means and standard deviations (± SD), while categorical variables were reported as frequencies and percentages. The mean value from multiple consecutive sleep studies (when applicable) was calculated for traditional OSA severity parameter (AHI events/h), sleep architecture (sleep stages percentage, WASO, and sleep efficiency), and hypoxic burden-derived variables. Differences were examined across OSA severity groups performing analysis of variance (ANOVA) between OSA classes.

#### Correlational analysis

To assess potential associations between emotion recognition during wakefulness and sleep parameters, Pearson’s correlation coefficients were computed. Based on these correlations, correlation matrices were built to identify variables with significant moderate-to-good associations with emotion recognition for further regression analysis (correlation coefficient *r* ≥ 0.4).

#### Visualization of the relation between sleep variables and emotion recognition

Data were calculated for tertiles of the variables of interest (according to significance level of Pearson’s correlation, hypothesis, and literature) and plotted graphs to detect trends and significance (ANOVA analysis across tertiles). Subsequently, we selected three representative variables to capture distinct aspects of sleep quality: “hypoxic burden” (indexed by desaturation severity), and sleep architecture (percentage of N1 stage, N1%, percentage of REM sleep, REM%). For each variable, tertiles were calculated, and participants were assigned points from 1 (the best tertile) to 3 (the worst tertile). The three scores were then summed to yield an overall “multidimensional sleep quality score” ranging from 3 (best possible sleep score) to 9 (worst possible sleep score). This composite score was subsequently used to visualize the relationship between sleep quality and decision-making latency (reaction time) for the emotion recognition stimuli.

#### Independent association between sleep and emotion recognition

**Figure 2:**
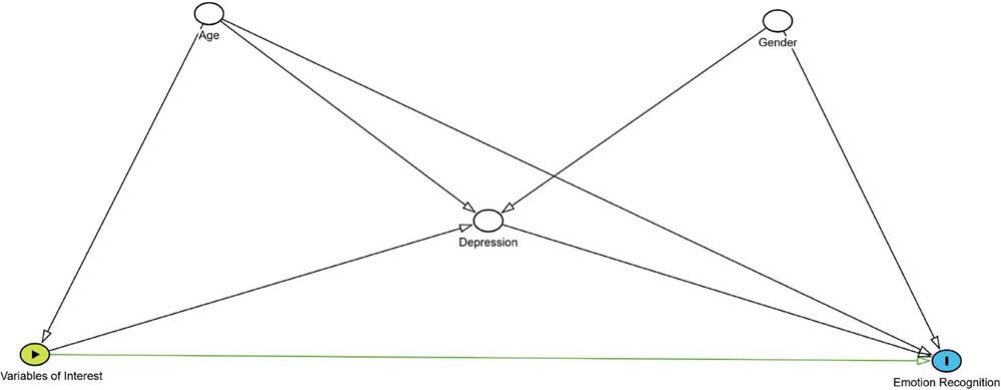
Directed acyclic graph (DAG) for illustration of the analysis rationale. Emotion recognition as the main studied outcome, sleep variables as the main exposures. Age, gender, and depression were used as covariates.

Selected variables and confounders in the regression analysis are shown in the explanatory-directed acyclic graph (Figure 2) according to the following rationale. The multidimensional sleep quality score may be associated with emotion recognition. Sleep quality may be affected by OSA or other sleep disturbances. Since age and gender strongly link with both OSA prevalence and cognitive function, we included both as confounders. Depression, expressed as DASS-21 depression scale score, is included as a mediator in a separate model, due to its interaction with age, gender, sleep quality, and the potential impairment in emotion recognition driven by depressive status. This allows for a distinction between the total effect, and the direct effect of the variable of interest on emotion recognition.

We pooled all different emotions (happiness, sadness, anger, fear) and performed separate analyses for the two intensity levels (high, low) and the neutral stimuli. Three different linear regression models were used to investigate the contribution of selected sleep variables (based on the above-mentioned correlational analysis) to reaction time and correctness of answers for high/low intensity and neutral emotions:

Model 1: Uncontrolled linear model, only the emotion intensity (as dependent variable) and the sleep variable (independent variable) are included.

Model 2: Controlled model with gender and age added as covariates (total effect model).

Model 3: Second controlled model with covariates of model 2, plus DASS-21 depression scale score.

#### Sensitivity analyses

Altogether, four sensitivity analyses were performed utilizing different subsets of the data, separated by: (1) gender, (2) sleep health status “healthy,” “insomnia only” (defined as Insomnia severity index (ISI > = 15)), “OSA only” (clinically relevant cut-off AHI ≥ 15) and “COMISA” (comorbid insomnia and sleep apnea, according to definitions above), and (3) the presence of Excessive Daytime Sleepiness (EDS, defined as Epworth Sleepiness Scale (ESS) with a cutoff >10). (4) Correlation and linear regression analyses were repeated using the PSG results from the first single night and compared to those from sleep results obtained by multiple recordings (*n* = 47).

All tests were evaluated as significant with a threshold of *p*-value <.05. Due to the explorative character of the study, no adjustment for multiple statistical testing was applied. Statistics, tables, and images were generated using R version 4.5.0.

### Ethical approval

All participants signed an informed consent form after receiving the relevant oral and written information, and the study was approved by the National Bioethics Committee and the Data Protection Authority of Iceland (VSN number 21–070).

## Results

### Anthropometric and clinical data

**Figure 3:**
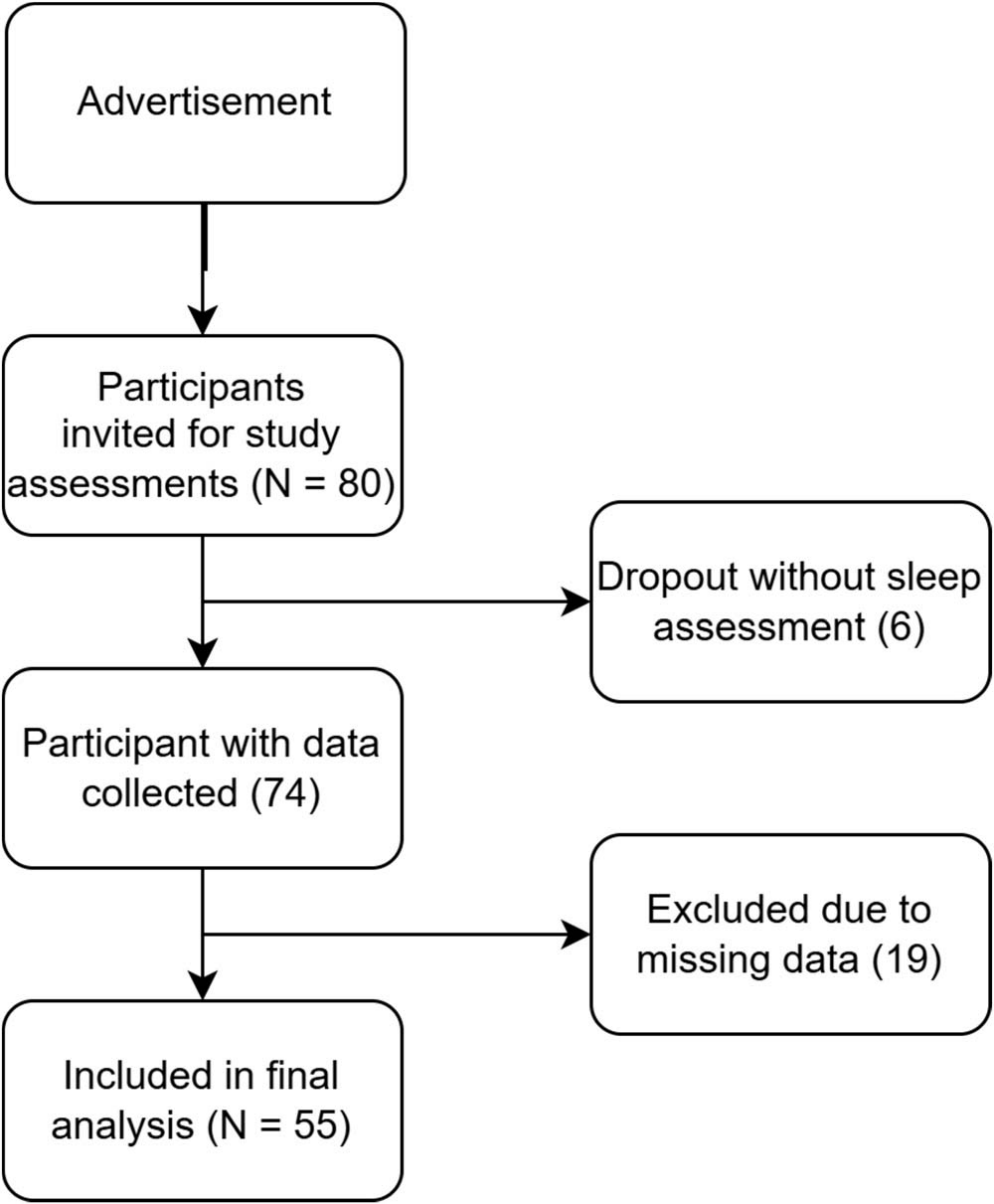
Study flow chart.

A total of 55 participants with a complete dataset were included in the study (Figure 3). Demographic and clinical characteristics show increasing age, BMI, and male gender distribution across OSA severity groups (Table 2).

**Table 2:** Anthropometric data, symptoms, objective sleep parameters, and crude ER-40 performance data

	Total study cohort	NO OSA (AHI < 5)	Mild OSA (5 ≤ AHI < 15)	Moderate OSA (15 ≤ AHI < 30)	Severe OSA (AHI ≥ 30)	*P*-value
*N*	55	19	16	13	7	
Male (%)	26 (47.3)	3 (15.8)	11 (68.8)	7 (53.8)	5 (71.4)	**.006**
Age	46.4 (14.4)	37.8 (14.0)	46.3 (13.2)	52.4 (9.0)	58.4 (14.5)	**.002**
BMI	27.9 (10.2)	25.4 (4.4)	27.8 (4.3)	30.4 (4.4)	30.9 (2.9)	**.004**
	Sleep parameters					
Total sleep time (h)	6.4 (0.9)	6.4 (1.1)	6.4 (0.7)	6.4 (1.0)	6.2 (0.5)	.969
N1%	8.0 (5.4)	5.4 (2.4)	7.2 (2.9)	8.6 (4.7)	15.9 (9.1)	**<.001**
N2%	42.6 (7.5)	42.9 (7.6)	44.5 (7.3)	41.0 (8.8)	40.5 (5.4)	.556
N3%	20.8 (9.1)	24.6 (9.9)	18.9 (7.6)	21.1 (8.3)	14.5 (8.8)	.059
NREM%	71.4 (4.3)	72.9 (4.6)	70.6 (4.1)	70.7 (3.6)	70.9 (4.8)	.361
REM%	20.2 (5.9)	21.6 (5.3)	21.1 (5.3)	19.8 (5.7)	15.3 (7.9)	.102
WASO (min)	34.6 (26.1)	21.8 (12.2)	33.4 (23.3)	40.0 (21.8)	62.0 (43.5)	**.003**
Arousal Index	14.7 (8.3)	10.8 (3.9)	12.6 (3.7)	15.3 (4.1)	29.1 (14.2)	**<.001**
Sleep Efficiency (%)	89.8 (6.1)	92.3 (3.8)	89.8 (5.8)	89.0 (5.1)	84.6 (10.0)	**.032**
ODI	13.6 (14.8)	2.5 (1.5)	8.0 (3.7)	19.3 (4.2)	45.6 (12.0)	**<.001**
AHI	15.1 (15.6)	2.4 (1.5)	9.0 (2.4)	23.7 (4.9)	47.6 (10.4)	**<.001**
Desaturation Severity	0.3 (0.6)	0.0 (0.0)	0.1 (0.1)	0.2 (0.2)	1.5 (1.2)	**<.001**
Desaturation Duration	5.5 (10.0)	0.3 (0.3)	1.6 (1.2)	6.0 (4.3)	27.1 (13.7)	**<.001**
Recovery Index	5.4 (10.3)	0.3 (0.3)	1.5 (1.2)	5.4 (3.7)	28.2 (13.0)	**<.001**
Recovery Severity	0.1 (0.2)	0.0 (0.0)	0.0 (0.0)	0.1 (0.1)	0.5 (0.4)	**<.001**
Recovery Duration	2.2 (3.8)	0.2 (0.2)	0.8 (0.6)	2.7 (1.9)	10.2 (5.0)	**<.001**
	ER-40					
Neutral intensity mean RT (ms)	3301 (2192)	2867 (1079)	2665 (1197)	3990 (2987)	4708 (3651)	.096
High intensity mean RT (ms)	2746 (1294)	2420 (874)	2689 (825)	2757 (776)	3739 (2893)	.146
Low intensity mean RT (ms)	2786 (995)	2617 (743)	2564 (846)	2687 (554)	3936 (1752)	**.009**
Neutral intensity correct answers (%)	85.0 (19.2)	90.8 (11.7)	84.4 (20.7)	84.6 (20.5)	71.4 (25.7)	.152
High intensity correct answers (%)	88.8 (8.9)	89.1 (8.8)	89.5 (8.5)	90.4 (7.9)	83.0 (11.3)	.329
Low intensity correct answers (%)	73.3 (10.2)	74.3 (9.8)	71.5 (9.4)	77.4 (9.0)	67.0 (13.4)	.139
	Questionnaires					
DASS21 depression	6.0 (6.3)	5.9 (5.7)	5.9 (5.9)	5.2 (7.0)	7.7 (8.1)	.873
ISI	13.1 (6.0)	8.1 (3.6)	13.6 (5.2)	16.4 (7.3)	14.9 (4.9)	.052
ESS	8.4 (4.8)	8.1 (3.6)	8.4 (4.9)	7.3 (5.0)	11.8 (7.2)	.295

Abbreviations: AHI = apnea hypopnea index, BMI = body mass index, DASS21 = Depression Anxiety Stress Scale 21 items, ESS = Epworth Sleepiness Scale, ISI = Insomnia Severity Index, N1% = percentage of N1 stage in total sleep time, N2% = percentage of N2 stage in total sleep time, N3% = percentage of N3 stage in total sleep time, NREM% = percentage of non-REM stage in total sleep time, ODI = Oxygen Desaturation Index, RT = reaction time, WASO = Wake time after sleep onset.

Mean and standard deviation for anthropometric, sleep, psychological, and ER-40 outcomes. Gender distribution is presented by number and percentage. Mean reaction time is calculated only from correct answers. *P*-value shows significant differences from ANOVA across the 4 OSA groups. Column “total study cohort ” represents descriptive statistics for the whole dataset, not included in the ANOVA.

### Sleep analysis

Sleep study data were based on three self-applied PSG nights in 30 cases, on two nights in 17 cases, and on a single-night assessment in eight cases. OSA was identified in 36 participants.

Sleep quality was significantly impaired across OSA severity groups for key metrics such as WASO, arousal index, and N1% (Table 2). The desaturation severity and duration, as well as recovery index, severity, and duration were all more pronounced in severe OSA. When examining emotion intensity levels (high, low) and neutral stimuli, only the mean reaction time for low-intensity stimuli differed between OSA groups (*p* = .009), with the worst performance in the severe OSA group (3936 ± 1752 ms) followed by moderate (2687 ± 5549 ms), healthy (2617 ± 743 ms), and mild OSA (2564 ± 846 ms) groups. In contrast, the analysis of DASS-21 revealed no significant differences across OSA groups for symptoms of depression.

### Correlations between ER-40 and sleep variables

**Figure 4:**
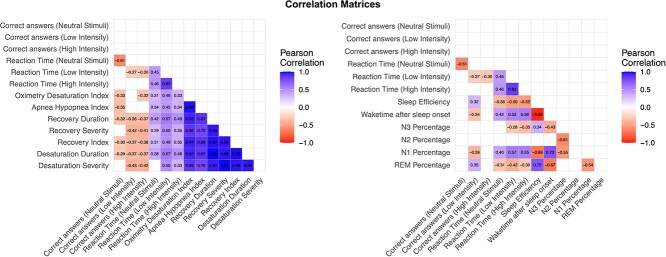
Heat map for correlation between emotion recognition and sleep/respiratory variables derived from polysomnography. Heatmap showing Pearson correlation with a significance level (*p* < .05) for different indices reflecting OSA severity, including hypoxia variables (A), and sleep architecture (B). Shown are results from Pearson correlation, empty boxes are for non-significant correlation. Colored boxes report Pearson correlation R-value both as a 3-digit number and as colored heatmap as in the legend on the right. Gray cells represent the diagonal elements (self-correlations) of the matrix. Abbreviations: N1 = percentage of N1 stage in total sleep time, N2 = percentage of N2 stage in total sleep time, N3 = percentage of N3 stage in total sleep time.

Figure 4, A shows the correlations between OSA variables derived from ABOSA and traditional PSG and different intensities (high and low) and neutral stimuli for emotion recognition. The correlations between sleep architecture variables and ER-40 stimuli are given in Figure 4, B. Only statistically significant outcomes are shown in the matrices with Pearson’s *r* correlation coefficient reported to two decimal places and presented in a gradient colored format. Most prominent variables correlating significantly with ER-40 outcomes were REM%, N1%, WASO, and sleep efficiency.

**Figure 5:**
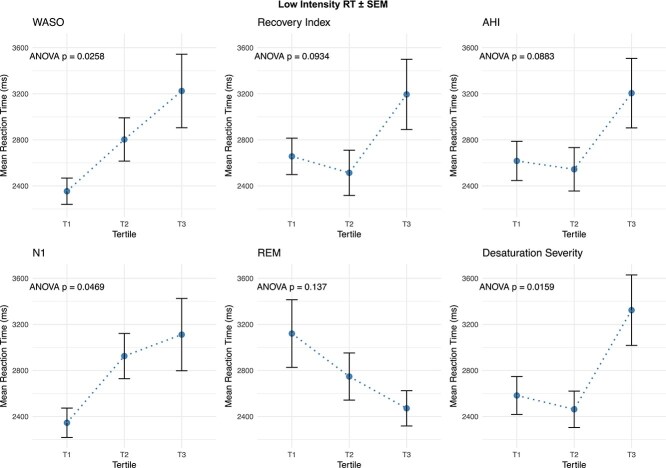
Mean reaction time during the ER-40 task in relation to tertiles of sleep quality and sleep apnea-related parameters. Tertile classification of prominent variables from the correlational analysis with mean reaction time (RT) in the ER-40. Shown are mean RT ± standard error of the mean (SEM) for each tertile. Abbreviations: AHI = apnea hypopnea index, N1% = percentage of N1 stage in total sleep time, REM = percentage of rapid eye movement stage in total sleep time, RT = reaction time (ms), SEM = standard error of the mean, T1 = first tertile, T2 = second tertile, T3 = third tertile, WASO = wake after sleep onset.

Following the correlational analysis, a tertile classification was performed to examine whether reaction times differed significantly across tertiles. The strongest associations between sleep quality measures/OSA indices and emotion recognition were observed for low intensity emotion stimuli (Figure 5), suggesting that an increase in reaction time was associated with poorer sleep architecture (i.e. higher WASO and greater N1%) and greater desaturation severity.

### Linear regression outcomes: emotion recognition predictors for reaction time and accuracy

#### Reaction time

Sleep variables that correlated significantly with emotion recognition outcome were entered into linear regression models to assess the stability of these associations while adjusting for age, gender, and symptoms of depression.

Across models, longer mean reaction times were significantly associated with greater hypoxic burden during sleep, particularly for the low-intensity emotion stimuli. Both hypoxic burden and sleep architecture-related variables (sleep stages, sleep efficiency) proved to be reliable predictors of reaction time for low-intensity emotion, while only sleep-architecture variables predicted high-intensity emotion performance (Table 3). Increased REM% and sleep efficiency were both associated with faster reaction times. No variables had a significant impact on reaction time for neutral stimuli after controlling for age, gender, and depression symptoms.

**Table 3:** Independent predictors of reaction time (ms) in the ER-40 test (high/low intensities and neutral stimuli)

	OSA severity	Hypoxic burden	Sleep architecture
**High intensity emotion** **mean reaction time (ms)**	AHI Estimate B = 81	Desaturation severity Estimate B = 174	**N1%** Estimate B = 438^*^
			REM% Estimate B = −158
	ODI Estimate B = 73	Recovery index Estimate B = 156	**WASO** Estimate B = 467^**^
			**Sleep Efficiency** Estimate B = −416^*^
**Low intensity emotion** **mean reaction time (ms)**	AHI Estimate B = 240	**Desaturation severity** Estimate B = 363^**^	**N1%** Estimate B = 409^**^
			**REM%** Estimate B = −310^*^
	ODI Estimate B = 254	**Recovery index** Estimate B = 328^*^	**WASO** Estimate B = 356^*^
			**Sleep Efficiency** Estimate B = −335^*^
**Neutral intensity emotion** **mean reaction time (ms)**	AHI Estimate B = 305	Desaturation severity Estimate B = 75	N1% Estimate B = 313
			REM% Estimate B = −301
	ODI Estimate B = 190	Recovery index Estimate B = 192	WASO Estimate B = 315
			Sleep Efficiency Estimate B = −345

Abbreviations: AHI = apnea hypopnea index, N1% = percentage of N1 stage in total sleep time, REM % = percentage of rapid eye movement sleep stage in total sleep time, ODI = Oxygen Desaturation Index, OSA = obstructive sleep apnea, WASO = Wake time after sleep onset.

Linear regression models for reaction time adjusting for age, gender, and the DASS-21 depression score. Estimated values (B) after standardization of the independent variable are shown, indicating how much the reaction time varies with an increase of 1 standard deviation of the explanatory variable. Bold and asterisks indicate the significance level: ^*^p < .05, ^**^p < .01, ^***^p < .001

Similarly, AHI did not predict reaction time after controlling for age, gender, and depression symptoms.

#### Accuracy

Similar to reaction time, hypoxic burden and sleep architecture-related variables significantly predicted response accuracy (Table 4), with enhanced performance associated with REM and sleep efficiency after controlling for age, gender, and symptoms of depression. AHI and ODI only remained significant in the model adjusted for age, gender, and symptoms of depression for neutral stimuli.

**Table 4:** Independent predictors of accuracy in the ER-40 test (high/low intensities and neutral stimuli)

	OSA severity	Hypoxic burden	Sleep architecture
**High intensity emotion**			
**% of correct answers**	AHI Estimate B = −2.0	**Desaturation severity** **Estimate B =** −3.3^*^	N1% Estimate B = −1.8
			REM% Estimate B = 0.5
	ODI Estimate B = −2.7	**Recovery index** Estimate B = −3.2^*^	WASO Estimate B = −0.5
			Sleep efficiency Estimate B = −0.2
**Low intensity emotion** **% of correct answers**	AHI Estimate B = −2.1	**Desaturation severity** **Estimate B =** −4.6^**^	**N1%** **Estimate B =** −5.0^**^
			**REM%** Estimate B = 3.6^*^
	ODI Estimate B = −2.8	**Recovery index** **Estimate B =** −4.1^*^	**WASO** **Estimate B =** −3.9^*^
			**Sleep efficiency** Estimate B = 3.4^*^
**Neutral intensity emotion** **% of correct answers**	**AHI** Estimate B = −6.7^*^	Desaturation severity Estimate B = −3.5	N1% Estimate B = −0.7
			REM% Estimate B = −0.9
	**ODI** **Estimate B =** −6.1^*^	Recovery index Estimate B = −4.9	WASO Estimate B = −1.2
			Sleep efficiency Estimate B = 1.3

Abbreviations: AHI = apnea hypopnea index, Des Sev = desaturation severity, N1% = percentage of N1 stage in total sleep time, REM % = percentage of REM stage in total sleep time, ODI = Oxygen Desaturation Index, OSA = obstructive sleep apnea, RI = recovery index, WASO = Wake time after sleep onset.

Linear regression models for accuracy adjusting for age, gender, and the DASS-21 depression score. The estimated values (B) after standardization of the independent variable are shown, indicating how much the accuracy (%correct answers) varies with an increase of 1 standard deviation of the explanatory variable. Bold and asterisks indicate the significance level: ^*^p < .05, ^**^p < .01, ^***^p < .001.

**Figure 6:**
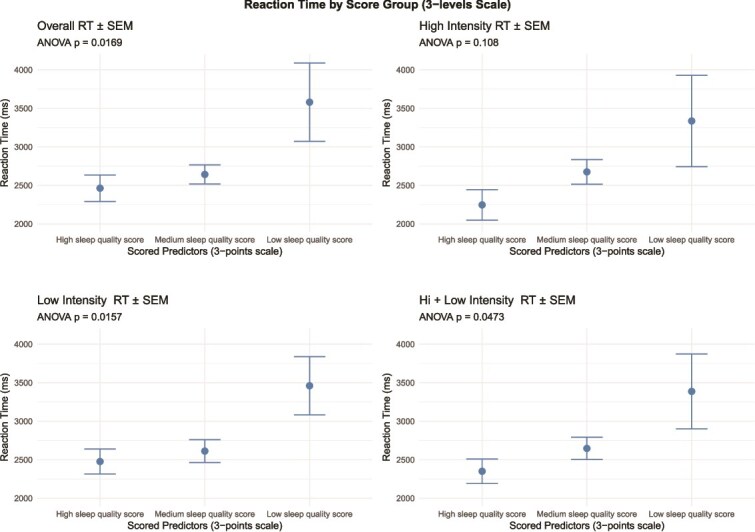
Mean reaction time during high and low intensity emotions in the ER-40 task in relation to the high, medium and low multidimensional sleep quality score. Abbreviations: RT = reaction time (ms), SEM = standard error of the mean. ANOVA = analysis of variance.

Following the correlation and the linear model analyses, we selected the three most representative variables to compute a multidimensional measure of sleep quality (see Materials and methods section). These three variables were N1%, REM%, and desaturation severity. Based on this calculation, the sleep quality score was grouped into high quality (3–4 points), medium quality (5–7 points), and low quality (8–9 points). As shown in Figure 6, reaction time increased as sleep quality decreased. The most pronounced and statistically significant effects were observed in the low-intensity condition, as well as in the combined mean of high and low intensity and in the overall intensity score (high, low, and neutral stimuli).

### Comparative predictive value of standard OSA vs. hypoxic burden metrics

Notably, across both latency and accuracy outcomes, the hypoxic burden-related metrics (i.e. desaturation severity and duration, recovery severity, duration and index) emerged more frequently as significant predictors in all models and for both high- and low-intensity emotion stimuli. In contrast, the standard OSA severity variables (AHI and ODI) were only significant predictors for the percentage of correct answers in the neutral stimuli condition.

### Sensitivity analyses

We identified significant gender differences with poorer sleep quality and higher OSA severity in males compared to females (Table S1). However, emotion recognition performance was comparable for males and females.

Individuals with good sleep health, insomnia only, OSA only or COMISA differed significantly in terms of clinical data, sleep quality, and OSA severity (Table S2). Individuals with COMISA showed significantly prolonged reaction time in emotion recognition, while the accuracy did not change.

Individuals with excessive daytime sleepiness (EDS) had a significantly lower REM% compared with the non-EDS group but no differences in ER-40 were found (Table S3).

Finally, we tested the hypothesis that sleep data from multiple nights would be superior in terms of predicting emotion recognition performance compared to parameters calculated based on a single night assessment. For this analysis, we used only participants who had at least two valid sleep studies (*n* = 47 participants, 45% male). Mean values showed no significant difference between the two groups (Table S4). However, the analysis revealed an increase in the percentage of variables that were significant predictors of emotion recognition for three nights compared with single night studies (Table 5).

**Table 5:** Comparison of significant predictors for emotion recognition using data from single night or means from multiple night PSG recordings

Mean reaction time						
	Single night			Multiple nights		
	High intensity	Low intensity	Neutral emotions	High intensity	Low intensity	Neutral emotions
Desaturation severity		**✔**			**✔**	
Desaturation duration		**✔**			**✔**	
Recovery index		**✔**			**✔**	
Recovery Severity		**✔**			**✔**	
Recovery duration					**✔**	
N1%				**✔**	**✔**	
REM%					**✔**	
Sleep efficiency				**✔**	**✔**	
ODI						
AHI						
WASO				**✔**	**✔**	
Arousal Index					**✔**	
Percentage of correct answers						
	Single night			Multiple nights		
	High intensity	Low intensity	Neutral emotions	High intensity	Low intensity	Neutral emotions
Desaturation severity	**✔**	**✔**		**✔**	**✔**	
Desaturation duration		**✔**		**✔**	**✔**	
Recovery index		**✔**		**✔**	**✔**	
Recovery severity		**✔**		**✔**	**✔**	
Recovery duration				**✔**	**✔**	
N1%		**✔**			**✔**	
REM%					**✔**	
Sleep efficiency		**✔**			**✔**	
ODI						**✔**
AHI						**✔**
WASO		**✔**			**✔**	
Arousal Index		**✔**			**✔**	

Abbreviations: AHI = apnea hypopnea index, Des Sev = desaturation severity, N1% = percentage of N1 stage in total sleep time, REM % = percentage of REM stage in total sleep time, ODI = Oxygen Desaturation Index, OSA = obstructive sleep apnea, RI = recovery index, WASO = Wake time after sleep onset.

The left columns (under single nights) are for variables from a single-night study, while the right columns are for variables derived from multiple (at least two) valid nights showing whether the variables were significant or not. Shown are results from Pearson correlation; empty boxes are for correlation with a non-reached significance level.

## Discussion

### Main findings

Our study provides evidence that emotion recognition, a highly important skill for social interaction and well-being, is strongly associated with different aspects of sleep quality. First, our results suggest that emotion recognition is associated with impaired sleep quality characterized by sleep disruption (higher N1%) and lower restorative function of sleep (lower REM%). Conversely, measures of sleep length were less prominent. Second, OSA severity predicted impaired emotion recognition. This association was stronger for hypoxic burden parameters than the traditional AHI used in clinical OSA severity classifications. Third, the study’s setting of a population-based sample highlights the general importance of sleep for emotion recognition. Even though OSA severity was rather mild and participants were almost ~10 years younger compared with typical clinical cohorts, a strong association between poor sleep quality and compromised emotion recognition was identified. Fourth, the data indicate that individuals fulfilling the definition for COMISA showed worse emotion recognition performance compared with each sleep disorder alone.

### Emotion recognition and OSA

Our work aligns with recent findings demonstrating that OSA is associated with several features related to facial emotion recognition. For example [6], reported that the typical advantage of healthy controls in classifying positive faces disappeared in individuals with OSA. More specifically, participants with OSA performed worse than the healthy control group in terms of emotion classification, showing a negative bias supported by the reduced speed of recognition of the negative emotion “sadness,” underlining the impact of OSA on emotion recognition. Similarly, children with moderate/severe OSA show a comparable lack of positive classification advantage with deficits in categorizing facial expressions when compared to non-apneic controls [24].

Additionally, our results show that impaired emotion recognition is associated with hypoxic burden and sleep architecture in relation to OSA. This finding aligns with prior work demonstrating that the neural mechanisms underlying emotion recognition deficits, characterized by increased reaction times and reduced accuracy, likely involve hypoxia-sensitive structural and functional key regions involved in emotion processing. In 2016, Tahmasian et al. documented grey-matter loss in limbic structures in OSA participants, particularly the basolateral amygdala and insula, which correlate with nocturnal hypoxemia severity. Complementary fMRI studies reveal improved functional connectivity between insular subregions and the whole brain in PAP (positive airway pressure) treated OSA patients [25], suggesting network-level compromise in hypoxic patients that may underline slow and inaccurate emotion recognition.

In the current study, hypoxic burden was associated with both higher reaction time and decreased accuracy for ER-40. Interestingly AHI did not predict reaction time and was a significant predictor only for neutral stimuli for accuracy not for high and low intensity emotion stimuli.

Most studies in the context of sleep apnea literature, focus on standard PSG parameters such as the AHI, ODI, or sleep stages determined after a single-night sleep study. Despite AHI being the gold standard parameter for diagnosis and severity estimation of OSA [26], the correlation between cognitive performance and AHI is generally weak [27] or absent [28]. The AHI is also only weakly associated with other sleep-apnea-related adverse events, such as cardiovascular disease-related mortality [29]. Hence there is a need to find better predictors. Alternative measures of OSA such as the hypoxic burden or the desaturation severities have been proposed as alternative metrics to reflect its association with comorbidity. These metrics assess not only the frequency, but also the duration and depth of desaturation events [17, 29–31]. Indeed, those parameters have been shown to better predict cognitive functions such as verbal memory and executive functioning [32], vigilance [19], sustained attention, and reaction time [33] compared with the traditional AHI measure.

In accord with knowledge on the impact of hypoxemia in specific brain areas related to emotion recognition we found that hypoxic burden metrics appear to be stronger predictors of the outcome of the ER-40 than traditional PSG indices of OSA severity. This result confirms earlier findings that hypoxic burden more accurately captures neurocognitive risk in OSA than AHI alone [19, 34]. Our data extend these observations by showing that higher desaturation severity and recovery index not only predict lower overall accuracy but also disproportionately slow responses under both high and low-intensity emotion conditions, regardless of the presented emotion type.

### Emotion recognition and sleep architecture

In the current study, sleep architecture also emerged as a significant predictor of emotion recognition performance: a higher percentage of REM sleep was associated with faster reaction times and improved accuracy, while N1 percentage and WASO increase predicted a worse performance on both emotion recognition accuracy and reaction time. Markers of sleep architecture, such as fragmented sleep and reduced REM sleep, have been linked to an impairment in declarative memory and veridical memory for emotional content [35]. Furthermore, sleep fragmentation and intermittent hypoxia in OSA have been shown to produce structural atrophy and hypoconnectivity in key emotion-processing regions (e.g., basolateral amygdala, insula), suggesting associated dysfunction of emotional, sensory, and limbic processes [8, 9]. The current results further align with the established role of REM in emotion regulation and memory consolidation [8, 9, 35, 36]. Preserving REM sleep may therefore mitigate some of the social-cognitive effects of OSA.

Our findings show a pattern of results that match with literature showing abnormal responses in an emotion recognition task found in several paradigms of disturbed sleep, including acute experimental sleep loss/deprivation/restriction [37–40], sleep disorders [7], and more specifically in insomnia [41].

### Combined analysis using a multidimensional sleep quality score

Intermittent hypoxemia, sleep fragmentation, and, more in general, sleep disorders most likely impact several and different brain regions instead of a single specific brain area. Indeed, our findings suggest that factors such as fragmented sleep and sleep-related hypoxic burden may both play key roles in impaired emotion recognition abilities. To further explore the multidimensional impact of sleep quality on emotion recognition performance, we applied a composite sleep quality score in the analysis, combining several sleep-related dimensions (N1%, REM sleep, and sleep-related hypoxia). Thereby, we could show that participants with lower overall sleep quality demonstrated longer reaction times, particularly in low-intensity conditions and when averaged across combined intensity levels, underscoring the adverse effect of poor sleep quality on cognitive-emotion processing.

It is important to note that participants fulfilling the clinical thresholds for combined “insomnia” and “sleep apnea,” often referred to as COMISA [42], showed significantly worse performance in reaction times for low-intensity and neutral emotions compared to other groups. This important novel finding supports previous data that COMISA has a stronger negative impact on clinically important outcomes like health-related quality of life, cognition, and cardiovascular disease, and warrants combined treatment approaches [42–45] and needs to be addressed further in future studies.

### Strengths and limitations

Important strengths of the study include the use of a population-based sample, increasing the generalizability of the findings. To control for possible inclusion bias, we identified a comparable distribution of sleep disorders in our cohort compared to large population-based studies in Iceland. Insomnia was present in 46% of our sample compared with 64% in a wider Icelandic population study [46], and OSA with 65% of our population versus 43% [47]. The cognitive test for emotion recognition, ER-40, is a well-known and validated tool for emotion recognition assessment with strong psychometric properties recommended for clinical use [48]. We applied the ABOSA software to retrieve accurate desaturation-derived parameters from overnight oximetry. Hypoxic load during sleep has already been demonstrated to be more reliable in relation to other aspects of cognitive functioning if compared to AHI [19, 32, 33]. The use of multiple consecutive nights of home self-applied PSG provided highly reliable sleep data. Indeed, means from multiple nights showed stronger associations with emotion recognition performance compared to single-night results. Our findings favor the use of multiple nights when identifying cognitive deficits of sleep disorders to mitigate first-night and night-to-night variability effects on sleep [14].

The most important limitation of the study was a limited sample size (*n* = 55), which did not allow us to address gender or age effects in more detail. Also, the influence of ethnicity or concomitant medication like antidepressants or hypnotics on the sleep-emotion recognition relationship could not be evaluated. The study design was cross-sectional, which did not allow for analysis of causation. Furthermore, our study did not address a possible treatment effect of sleep disorders on emotion recognition performance. The ER-40 provided stable and well-established normative data in the general population. However, facial emotion recognition in real life is not limited to fixed, static pictures, but relies on more complex factors [49]. Furthermore, using ER-40 in longitudinal pre-post treatment designs may offer valuable insights into the possibilities to reverse long-term effects of OSA-derived hypoxia.

### Clinical implications and future research

Our work adds facial emotion recognition to the spectrum of sleep-related cognitive vulnerabilities. The study highlights that chronic sleep fragmentation and hypoxic burden extend cognitive impairments beyond “cold” cognitive domains (e.g. attention, memory, executive function) [3, 19, 32, 33, 50] into “hot” affective processes.

Future research is needed to improve the validity of the tested paradigm. Significant importance should be placed on moving and testing the research setting to both a broad general population and clinical populations that is properly representative of OSA patients seeking treatment for their disease. Moreover, evaluating the effect of a pre-post treatment research design should be a focus for future studies on this specific topic.

### Summary

In summary, the severity of hypoxic load during sleep, sleep fragmentation, sleep continuity (evaluated using N1%), and REM%, are all independent predictors of emotion recognition performance in adults from the general population. The effects appear to occur already in mild OSA and an early, often preclinical state of disease trajectory. Importantly, hypoxic burden parameters produce more reliable predictors when compared with standard sleep apnea classification. The protective role of REM sleep and preserved sleep architecture for both accuracy and reaction time underscores the complexity in emotional processing. Our data are highly supportive of existing neurocognitive models of sleep-dependent affective regulation.

## Supplementary Material

Ferretti_D_Impaired_emotion_recognition_Suppl_Material_zsag078

## Data Availability

The data include sensitive medical information and, therefore, is not made publicly available.
