# Supplementary material for: Impaired emotion recognition is associated with sleep-related hypoxic burden
Source: Sleep. 2026 May 28;49(7):zsag078. doi: 10.1093/sleep/zsag078 (PMC13357497; doi:10.1093/sleep/zsag078)
Supplement: Ferretti_D_Impaired_emotion_recognition_Suppl_Material_zsag078 [file ferretti_d_impaired_emotion_recognition_suppl_material_zsag078.docx]

**SUPPLEMENTARY MATERIALS**

**Impaired emotion recognition is associated with sleep-related hypoxic burden**

*Dimitri Ferretti^1,2,3^, Elena Richert^1,2,4^, Ding Zou^3^, Jan Hedner^3,5^, Christian Strassberger^3^, Erna Sif Arnardottir^2,6,7^, Ludger Grote^3,5,*^, Kamilla Rún Jóhannsdóttir^1,2,*^*

*^1^Department of Psychology, Reykjavik University, Reykjavik, Iceland, ^2^Reykjavik University Sleep Institute, Reykjavik University, Reykjavik, Iceland, ^3^Department of Internal Medicine and Clinical Nutrition, Sahlgrenska Academy, University of Gothenburg, Gothenburg, Sweden,  ^4^Department of Technical Physics, University of Eastern Finland, Kuopio, Finland. ^5^Pulmonary Department, Sahlgrenska University Hospital, Gothenburg, Sweden, ^6^Department of Computer Science, Reykjavik University, Reykjavik, Iceland, ^7^Department of Engineering, Reykjavik University, Reykjavik, Iceland.
* Co-senior authors*

*Corresponding Author:
Dimitri Ferretti, Centre for Sleep and Vigilance Disorders, Sahlgrenska Academy, University of Gothenburg, Medicinaregatan 3, 41390, Gothenburg, Sweden. Email:* [*dimitri.ferretti@gu.se*](mailto:dimitri.ferretti@gu.se); [*dimitrif@ru.is*](mailto:dimitrif@ru.is)

**Results**

*Sensitivity analyses: Emotion recognition in different conditions*

In the presented supplementary material we show the results from our sensitive analysis performed to give a better understanding of data cohort.

Analysis for subgroups of participants have been performed to evaluate ER-40 results in more detail. In particular, results are presented for

- Individuals of male and female sex (Table S1),
- Participants classified as healthy sleepers, as individuals with OSA, insomnia or combined OSA and insomnia (COMISA) (Table S2),
- Participants with or without excessive daytime sleepiness defined by the Epworth Sleepiness Scale sore (Table S3), and
- Analysis based on results from single or multiple PSG night assessments (Table S4).

Presentation of the emotion recognition performance in individuals separated for gender.

**Table S1: Data presenting emotion recognition results for participants stratified by Sex.**

|  | female | male | p test |
| --- | --- | --- | --- |
| n | 29 | 26 |  |
| Age | 45.1 (13.1) | 47.7 (16.0) | 0.517 |
| BMI | 27.6 (5.1) | 28.3 (4.1) | 0.584 |
| Sleep Parameters | | | |
| Total Sleep Time (hours) | 6.6 (0.9) | 6.1 (0.7) | **0.049** |
| N1% | 6.2 (2.8) | 10.0 (6.8) | **0.008** |
| N2% | 43.3 (7.3) | 41.9 (7.9) | 0.513 |
| N3% | 21.9 (8.6) | 19.7 (9.7) | 0.347 |
| NREM% | 71.4 (4.5) | 71.5 (4.1) | 0.916 |
| REM % | 21.7 (5.8) | 18.6 (5.8) | 0.055 |
| Sleep Efficiency | 91.4 (5.3) | 88.0 (6.5) | **0.041** |
| ODI | 9.0 (10.2) | 18.7 (17.4) | **0.013** |
| AHI | 10.1 (12.0) | 20.6 (17.4) | **0.011** |
| WASO | 29.2 (21.7) | 40.6 (29.5) | 0.106 |
| Arousal Index | 12.1 (4.1) | 17.6 (10.6) | **0.013** |
| Desaturation Severity | 0.1 (0.2) | 0.5 (0.9) | **0.037** |
| Desaturation Duration | 2.6 (4.8) | 8.6 (13.0) | **0.024** |
| Recovery Index | 2.5 (4.67) | 8.6 (13.3) | **0.025** |
| Recovery Severity | 0.0 (0.1) | 0.1 (0.3) | **0.039** |
| Recovery Duration | 1.1 (2.0) | 3.4 (4.8) | **0.026** |
| ER-40 | | | |
| Neutral intensity  mean Reaction Time (ms) | 3388 (2156) | 3219 (2272) | 0.779 |
| High intensity  mean Reaction Time (ms) | 2485 (727) | 3036.9 (1690) | 0.115 |
| Low intensity  mean Reaction Time (ms) | 2580 (688) | 3015 (1227) | 0.106 |
| Neutral intensity  correct answers Percent | 83.19 (21.5) | 87.02 (16.4) | 0.464 |
| High intensity  correct answers Percent | 90.95 (8.5) | 86.30 (8.8) | 0.051 |
| Low intensity  correct answers Percent | 74.14 (9.7) | 72.36 (10.9) | 0.524 |
| Questionnaires | | | |
| DASS - Depression | 5.66 (6.0) | 6.31 (6.6) | 0.703 |

Abbreviations: AHI = apnea hypopnea index, BMI = body mass index, DASS = depression anxiety stress scale, ESS = Epworth Sleepiness Scale, ISI = Insomnia Severity Index, N1 = percentage of N1 stage in total sleep time, N2 = percentage of N2 stage in total sleep time, N3 = percentage of N3 stage in total sleep time, Non-REM % = percentage of non REM stage in total sleep time, Oximetry ODI = Oxygen Desaturation Index from oximetry, RT = reaction time, WASO = Wake time after sleep onset.

**Table S2: Results of the ER-40 test in individuals separated for no sleep disorder (healthy), Insomnia only, OSA only and COMISA as combined OSA and insomnia diagnosis.**
Table with participants stratified by Comorbid insomnia and obstructive sleep apnea (COMISA). Insomnia Severity Index cutoff of ISI >=15, and AHI cutoff for OSA set to AHI >=15. N = 50, 5 participants of the analyzed cohort were not included because did not fill the ISI questionnaire.

|  | Healthy | Insomnia Only | OSA Only | COMISA | p-value |
| --- | --- | --- | --- | --- | --- |
| N | 22 | 12 | 5 | 11 |  |
| male % | 6 (27.3) | 7 (58.3) | 3 (60.0) | 7 (63.6) | 0.132 |
| Age | 45.5 (14.2) | 34.2 (11.6) | 50.6 (6.5) | 59.1 (10.9) | **< 0.001** |
| BMI | 26.3 (3.8) | 26.7 (5.7) | 32.1 (3.4) | 29.5 (4.1) | **0.026** |
| Sleep Parameters | | | | | |
| Total Sleep Time (hours) | 6.5 (0.9) | 6.1 (0.7) | 6.8 (0.6) | 6.1 (0.9) | 0.268 |
| Desaturation Severity | 0.0 (0.0) | 0.0 (0.0) | 0.3 (0.2) | 1.1 (1.1) | **< 0.001** |
| Desaturation Duration | 0.8 (1.1) | 0.9 (0.9) | 8.2 (5.4) | 19.7 (14.9) | **< 0.001** |
| Recovery Index | 0.7 (1.0) | 0.9 (1.1) | 8.6 (6.6) | 19.5 (15.3) | **< 0.001** |
| Recovery Severity | 0.0 (0.0) | 0.0 (0.0) | 0.1 (0.1) | 0.3 (0.3) | **< 0.001** |
| Recovery Duration | 0.4 (0.6) | 0.4 (0.4) | 3.5 (2.2) | 7.6 (5.3) | **< 0.001** |
| N1 % | 6.3 (2.3) | 6.0 (3.6) | 11.1 (5.6) | 12.9 (8.7) | **0.002** |
| N2 % | 43.2 (6.3) | 43.9 (9.5) | 42.6 (10.5) | 41.0 (7.3) | 0.828 |
| N3 % | 21.2 (9.2) | 23.8 (9.8) | 16.0 (7.2) | 17.3 (9.1) | 0.25 |
| Non-REM % | 70.7 (4.4) | 73.7 (4.3) | 69.6 (3.7) | 71.2 (4.5) | 0.192 |
| REM % | 22.3 (5.2) | 19.8 (5.2) | 20.9 (3.6) | 16.0 (6.4) | **0.023** |
| Sleep Efficiency | 91.0 (4.1) | 91.3 (6.5) | 89.3 (4.2) | 85.5 (8.5) | 0.07 |
| WASO | 27.1 (12.9) | 26.6 (27.7) | 41.5 (14.9) | 56.1 (37.6) | **0.011** |
| Arousal Index | 11.9 (3.6) | 11.5 (4.5) | 20.1 (5.2) | 22.6 (13.8) | **0.001** |
| AHI (events/hours) | 4.9 (3.7) | 5.6 (3.6) | 32.0 (9.6) | 35.4 (16.0) | **< 0.001** |
| Oximetry ODI | 4.6 (3.7) | 5.2 (3.9) | 26.1 (8.0) | 34.0 (17.2) | **< 0.001** |
| ER-40 | | | | | |
| High intensity correct answers Percent | 90.3 (8.6) | 87.5 (8.8) | 90.0 (8.4) | 85.2 (10.6) | 0.463 |
| Low intensity correct answers Percent | 73.9 (10.5) | 71.9 (8.2) | 80.0 (9.3) | 67.6 (10.0) | 0.12 |
| Neutral intensity correct answers Percent | 88.07 (14.2) | 87.5 (21.3) | 80.0 (24.4) | 77.3 (22.9) | 0.417 |
| High intensity mean Reaction Time (ms) | 2595 (992) | 2419 (568) | 2860.6 (711) | 3611 (2266) | 0.133 |
| Low intensity mean Reaction Time (ms) | 2681 (771) | 2276 (552) | 2946 (634) | 3475 (1535) | **0.03** |
| Neutral intensity mean Reaction Time (ms) | 2873 (1170) | 2511 (1044) | 3771 (2498) | 5038 (3794) | **0.027** |
| Questionnaires | | | | | |
| DASS – Depression | 4.0 (4.1) | 9.7 (6.7) | 5.6 (6.1) | 7.1 (8.7) | 0.091 |

*Abbreviations: AHI = apnea hypopnea index, BMI = body mass index, DASS = depression anxiety stress scale, ESS = Epworth Sleepiness Scale, ISI = Insomnia Severity Index, N1 = percentage of N1 stage in total sleep time, N2 = percentage of N2 stage in total sleep time, N3 = percentage of N3 stage in total sleep time, Non-REM % = percentage of non REM stage in total sleep time, Oximetry ODI = Oxygen Desaturation Index from oximetry, RT = reaction time, WASO = Wake time after sleep onset.*

**Table S3: Table with participants stratified by EDS (Excessive Daytime Sleepiness) defined by Epworth Sleepiness Scale score > 10, N = 54.**

|  | | No EDS | EDS | p-value |
| --- | --- | --- | --- | --- |
| N | | 39 | 15 |  |
| male % | | 21 (53.8) | 5 (33.3) | 0.295 |
| Age | | 46.2 (14.8) | 45.0 (12.5) | 0.786 |
| BMI | | 28.0 (4.5) | 27.8 (5.3) | 0.862 |
|  | Sleep Parameters | | | |
| Total Sleep Time (hours) | | 6.4 (0.9) | 6.3 (0.9) | 0.659 |
| Desaturation Severity | | 0.2 (0.5) | 0.4 (1.0) | 0.289 |
| Desaturation Duration | | 4.5 (8.7) | 6.9 (12.7) | 0.439 |
| Recovery Index | | 4.2 (7.8) | 7.7 (14.5) | 0.25 |
| Recovery Severity | | 0.1 (0.1) | 0.1 (0.3) | 0.308 |
| Recovery Duration | | 1.9 (3.3) | 2.7 (4.7) | 0.451 |
| N1 % | | 8.1 (5.8) | 7.9 (4.8) | 0.934 |
| N2 % | | 41.8 (6.7) | 45.1 (9.3) | 0.146 |
| N3 % | | 21.1 (8.7) | 19.4 (10.4) | 0.56 |
| Non-REM % | | 70.9 (4.4) | 72.5 (4.0) | 0.226 |
| REM % | | 21.3 (5.8) | 17.6 (5.9) | **0.042** |
| Sleep Efficiency | | 90.1 (6.0) | 88.9 (6.5) | 0.518 |
| WASO | | 32.7 (25.9) | 40.3 (27.3) | 0.345 |
| Arousal Index | | 14.2 (7.4) | 16.2 (10.7) | 0.421 |
| AHI (events/hours) | | 13.7 (13.4) | 17.45 (20.2) | 0.425 |
| Oximetry ODI | | 12.0 (12.2) | 16.2 (19.8) | 0.347 |
|  |  | ER-40 | |  |
| High intensity correct answers Percent | | 88.9 (9.0) | 88.3 (9.1) | 0.825 |
| Low intensity correct answers Percent | | 73.1 (10.2) | 73.8 (11.4) | 0.833 |
| Neutral intensity correct answers Percent | | 87.5 (15.2) | 78.3 (26.9) | 0.12 |
| High intensity mean Reaction Time (ms) | | 2876 (1481) | 2377 (542) | 0.211 |
| Low intensity mean Reaction Time (ms) | | 2835 (1127) | 2582.0 (478) | 0.407 |
| Neutral intensity mean Reaction Time (ms) | | 3103 (1951) | 3785 (2793) | 0.314 |
|  | Questionnaires | | | |
| DASS – Depression | | 5.7 (6.5) | 6.5 (6.0) | 0.685 |

*Abbreviations: AHI = apnea hypopnea index, BMI = body mass index, DASS = depression anxiety stress scale, ESS = Epworth Sleepiness Scale, ISI = Insomnia Severity Index, N1 = percentage of N1 stage in total sleep time, N2 = percentage of N2 stage in total sleep time, N3 = percentage of N3 stage in total sleep time, Non-REM % = percentage of non REM stage in total sleep time, Oximetry ODI = Oxygen Desaturation Index from oximetry, RT = reaction time, WASO = Wake time after sleep onset.*

**Table S4: Comparison table for sleep variables between single and multiple nights.**

|  | Single Night | Multiple Nights |  |
| --- | --- | --- | --- |
| N | 47 | 47 | p-value |
| Total Sleep Time (hours) | 6.5 (1.1) | 6.4 (0.8) | 0.769 |
| Desaturation Severity | 0.2 (0.5) | 0.2 (0.6) | 0.75 |
| Desaturation Duration | 4.1 (7.5) | 4.5 (8.5) | 0.807 |
| Recovery Severity | 0.1 (0.1) | 0.1 (0.2) | 0.715 |
| Recovery Duration | 1.7 (2.7) | 1.9 (3.1) | 0.781 |
| N1 % | 7.5 (4.2) | 7.3 (4.2) | 0.837 |
| N2 % | 42.8 (7.9) | 43.1 (7.4) | 0.84 |
| N3 % | 21.5 (9.2) | 21.2 (8.8) | 0.879 |
| Non-REM % | 71.8 (5.6) | 71.7 (4.3) | 0.888 |
| REM % | 20.6 (6.3) | 20.4 (5.2) | 0.884 |
| Sleep Efficiency | 90.4 (5.1) | 90.3 (4.7) | 0.918 |
| WASO | 30.5 (19.0) | 32.4 (20.2) | 0.637 |
| Arousal Index | 14.5 (7.1) | 14.2 (7.5) | 0.863 |
| AHI | 14.1 (13.9) | 13.8 (14.1) | 0.92 |
| Oximetry ODI | 12.4 (12.8) | 12.5 (13.3) | 0.964 |

*Abbreviations: AHI = apnea hypopnea index, N1 = percentage of N1 stage in total sleep time, N2 = percentage of N2 stage in total sleep time, N3 = percentage of N3 stage in total sleep time, Non-REM % = percentage of non REM stage in total sleep time, Non-REM % = percentage of REM stage in total sleep time, Oximetry ODI = Oxygen Desaturation Index from oximetry, WASO = Wake time after sleep onset.*
